# Research into the correlation between positional skull deformation and motor performance of infants aged under 4 months

**DOI:** 10.1186/s12887-023-03959-6

**Published:** 2023-05-04

**Authors:** Tianqi Huang, Wenzao Li, Chengju Wang, Fuxiang Qu, Qiuxia Yang, Qiuming Pan, Xiaoqin Pu, Can Xiao, Yi Cai, Meifeng Xia, Yuping Zhang

**Affiliations:** Department of Pediatrics, the Second Affiliate Hospital of Army Medical University, No. 83 Xinqiao Street, Chongqing, 400037 China

**Keywords:** Positional skull deformation, The infant motor performance test, Motor performance of infants in early infancy

## Abstract

**Objective:**

To investigate the correlation between positional skull deformation (PD) and motor performance of infants under 4 months of age.

**Methods:**

Infants aged under 4 months were enrolled in the children’s healthcare and the premature infants follow-up Clinic of the Second Affiliated Hospital of Army Military Medical University. The cranial vault asymmetry (CVA) and cephalic index (CI) were calculated in all infants, and the infant motor performance test (TIMP) was used to evaluate the infant motor performance. The motor performances of infants with different types and degrees of PD were compared, so were the incidences of PD in infants with different motor performance levels.

**Results:**

Overall, 2118 infants were recruited and divided according to the types of PD and TIMP scores. The comparison of TIMP scores within different types of PD at different months of age showed that, regardless of the types of PD, TIMP scores of infants with PD were lower than those of normal infants. In particular, the difference in TIMP scores was statistically significant (*P* < 0.05) in infants with dolichocephaly, plagiocephaly,dolicho-plagiocephaly and brachy-plagiocephy. In addition, the comparison of CVA values of infants with different TIMP score levels at different months of age showed that the CVA values of the extremely low-level group were significantly higher than those of the medium-level and high-level group, especially in the 3-month-old and 4-month-old groups, which showed significant statistical differences (*P* < 0.05).

**Conclusions:**

PD and motor performance of infants aged under 4 months seem to interact and influenc each other. The more serious the severity of PD were,the worse the motor performance of infants. Conversely, the incidence of PD increased in infants with poor motor performance.

## Background

Positional skull deformation (PD) is a condition in which the shape of the skull deforms as a result of prolonged extrinsic compression during the early stage of infancy [1, 2], and it was also known as deformational plagiocephaly (DP), deformational brachycephaly(DB) or positional plagiocephaly(PP) [3–5]. PD which can be divided into plagiocephaly, brachycephaly,dolichocephaly, brachy-plagiocephaly and dolicho-plagiocephaly [6], is related to the sleep positioning remain, premature birth, low birth weight and other factors [3]. It has been reported that with the advent of the American Academy of Pediatrics’*Back to Sleep campaign* since 1990s, a notable side effect of the change in policy has been a dramatic rise in the incidence of skull asymmetry among infants [7, 8]. It was of increasing interests in the studies of PD among developed countries, and some of the studies reported that PD would impact the motor development [9, 10], while many professionals believed that PD mainly affected the appearance of the skulls and was not involved with the motor development [11].

In the first few months of life which is with the highest incidence of PD, the peak prevalence is at 4 months of age [12, 13]. Therefore, this stage is the best age phase for the study of the correlation between cranial deformation and development of infants. But It is difficult to evaluate the motor performance as the infant has little active positioning of head and torso at this age stage, which brings difficulties to the research. However, the Test of Infant Motor Performance (TIMP) provides a feasible option. TIMP originally created by Girolami in 1983, is an internationally recognized scale for the assessment of early motor development in infants, which also has been updated to the fifth edition and is used in more than 40 countries and regions around the world. TIMP can be used both with infants born on time and with infants born prematurely between the 34th weeks of postmenstrual age and the 4 week postterm (age adjusted for prematurity if necessary). The infant's spontaneous movements and the infant’s movement responses to various positions and to sights and sounds are tested through observed items and elicited items in order to identify infants with delayed functional motor performance [14, 15]. Hence, the application of TIMP in the early motor assessment of infants with PD is practicable.

In clinical work, we often see poor motor performance in infants with severe PD, so we believe that there may be a correlation between PD and infant motor development. Therefore, we conducted the TIMP assessment while measuring the craniotype of infants under 4 months of age to investigate the effects of different types and degrees of PD on motor performance. And further exploration was performed into the influence of different motor performance levels on skull shape as to trying to get the clues of the controversial questions from the clinical data of early life stage: Does PD in early infancy have an effect on motor performance? Are poor motor skills in early infancy correlated with PD? What is the correlation between PD and motor retardation?

## Subjects and method

### Subjects

A total of 2118 infants under age of 4 months old (age adjusted for prematurity) were recruited for study from the children’s healthcare and the premature infants follow-up Clinic of the Second Affiliated Hospital of Army Military Medical University between 31th July 2018 and 4th December 2019.Healthy infants were considered to be eligible for the study and the exclusion criteria were as follows: 1) Children with PD caused by congenital torticollis and synostosis; 2) Children with PD having received any type of therapy for PD; 3) Parents refusing to participate in this study. This research was approved by the ethics committee of the Second Affiliated Hospital of Army Military Medical University, and all the guardians of the selected subjects signed informed consent before the study was carried out. The Consent for testing was provided by physicians, and informed written consent was obtained from the infants’ parents or primary caregivers.

### Method 

#### Collection of general information

The general information of the infants were collected, including gender, date of birth, birth weight, gestational age at birth, mode of delivery, etc.

#### Measurement of skull shape and calculation of derived value

**Fig. 1:**
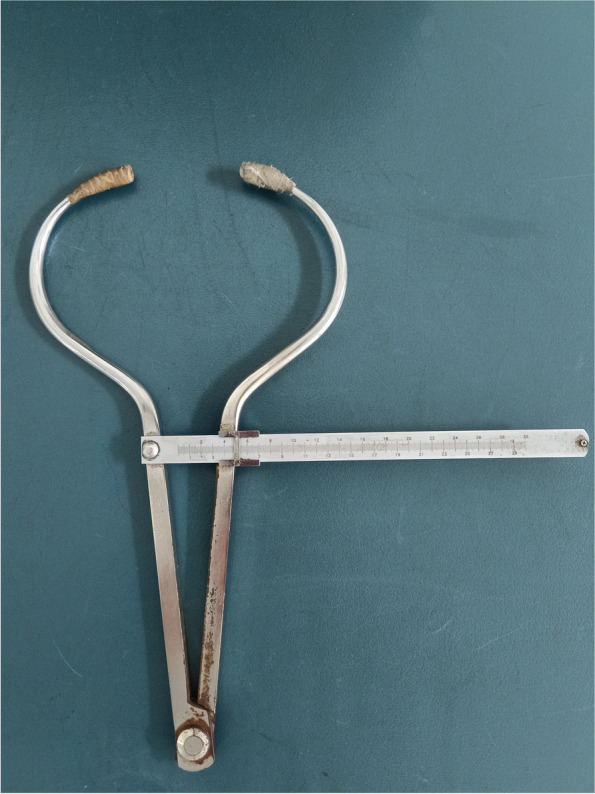
The measurement tool was the KWJ124 bending foot gauge (size 260 × 260 + 36 mm),the measurement range was 0–300 mm, and the executive production standard was GB5704.3–85

**Fig. 2:**
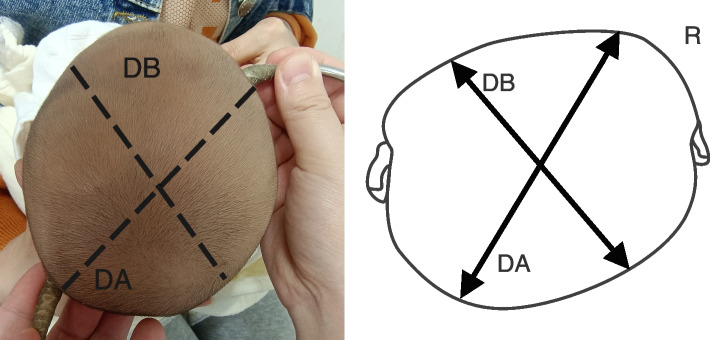
Transcranial oblique diameter is the distance from the middle point of the temporal ridge of frontal bone to the inner edge of the contralateral herring bone suture; the long diameter is diagonal A (DA); the short diameter is diagonal B (DB)

**Fig. 3:**
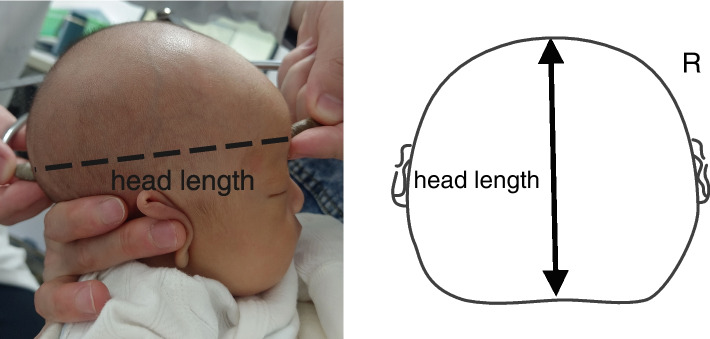
Head length is the distance from the glabella to the farthest point (opisthocranion, op)

**Fig. 4:**
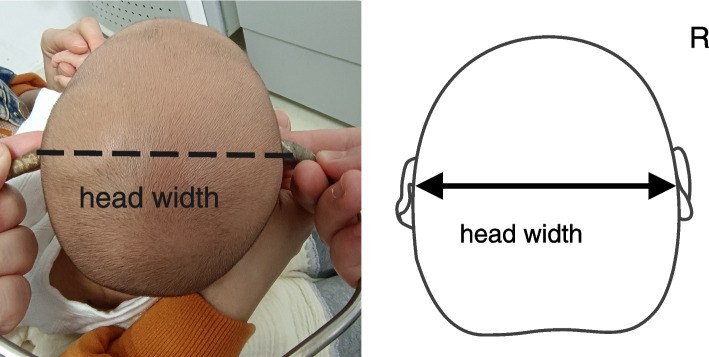
Tead width is the distance between two points 1 cm higher than the attachment point of both ears

The manual measurement method of Wilbrand et al.’s standardization scheme was adopted. Measurement tools and specific measurement methods are shown in Figs. 1, 2, 3, and 4. All measuring lines were parallel to the Frankfurt line.According to the method of standard measurement scheme,we can get the values of diagonal A (DA),diagonal B (DB),head length and head width.Using these values, the following were calculated: CVA = difference of the oblique diameter on both sides of the head (CVA = DA-DB), in mm; CI = ratio of the maximum transverse diameter of the cranial to the maximum fore-and-aft diameter (CI = cranial width/cranial length × 100%) [16]. According to the reliability test, the measurement difference among survey personnel was less than 5%.

#### Test of infant motor performance

The examination was carried out by physical therapists competent to assess the motor condition of infants who were also trained according to the methodological requirements for performing the TIMP test (the 5th edition) in the presence of parents. The infants who were in a quiet, alert status were placed on a plane wearing diapers or less clothing in a quiet, bright and warm room. The whole evaluation process lasted about 15 to 30 min, in which there were 42 items. Each item should be tested successfully once as far as possible, and repeated no more than 3 times at most. If the test was interrupted, the remaining items had to be completed within 24 h. The first 13 items were observed items, and the infant would get 1 point if it appeared the corresponding performance, otherwise, it would get 0. The last 29 items were elicited items, which were divided into different grades according to different motor performance of the infant. The score range was 0 to 6 points, and the total score was obtained by adding the scores of 42 items. The motor performance level of the infant was deemed by the percentile of the total score in the norm percentile curve of the week-old age group. With a cross-sectional study design, infants were examined only once [17].

#### Grouping of subjects

##### Grouping by types of skull deformation

Diagnostic criteria were based on the recommended diagnostic criteria of positional skull deformation in infants aged 0 to 6 months in Chongqing area [18]. Infants of different ages were grouped according to different types and degrees of PD (Table 1).

**Table 1:** Diagnostic criteria of type and severity of positional skull deformity

	Plagiocephaly(CVA)	Brachycephaly(CI)	Dolichocephaly(CI)
Mild	4–6.9 mm	91–95%	79–82%
Moderate	6.9-10 mm	95–99%	76–79%
Severe	≥ 10 mm	≥ 99%	≤ 76%

*CVA* cranial vault asymmetrym,normal range is 0-4 mm, *CI* cephalic index,normal range is 82–91%

##### Grouping by TIMP Scores

The infants were grouped according to the TIMP scores, the standard of which was based on the TIMP norm percentile curve of China (based on unpublished data in the research department). Infants of different month ages with total TIMP scores lower than the 10th percentile (P10) were extremely low-level group; P10-P25 was low-level group; P25-P75 was medium-level group and P75 or more was high-level group.

### Statistical analysis

Statistical analysis was performed by using IBM SPSS Statistics 23.0 for Windows (IBM Germany GmbH). Measurement data were represented as mean ± standard deviation, and the mean values between the two groups were compared by one way ANOVA. Counting data were expressed as frequency or frequency percentage, and cross-table Chi-square test was used for comparison between the two groups. *P* < 0. 05 were considered statistically significant.

## Results

### Patient characteristics

A total of 2118 infants were enrolled in this research including 1110 males and 1008 females. General characteristics of each group are shown in Table 2. There were differences in gestational age and birth weight between Infants with PDs and normal infants, among which plagiocephy, brachycephaly and brachy-plagiocephy infants were the most significant, while there were no significant differences in gender and delivery style.

**Table 2:** Patient characteristics

Month age		Normal	Plagiocephaly	Brachycephaly	Dolichocephaly	Brachy-plagiocephaly	Dolichol-plagiocephaly
0	Gender (male / female, n)	43/41	39/32	5/8	74/71	15/11	30/20
	Gestational age (weeks, X ± s)	37.47 ± 2.34	36.50 ± 2.72^*^	35.05 ± 3.68^*^	37.86 ± 1.51	34.04 ± 3.45^*^	37.40 ± 1.82
	Birth weight (kg, X ± s)	2.88 ± 0.66	2.62 ± 0.73^*^	2.29 ± 0.79^*^	3.01 ± 0.53	2.25 ± 0.90^*^	2.78 ± 0.68
	Birth mode (natural birth / cesarean section, n)	37/47	38/33	9/4	64/81	12/14	26/24
1	Gender (male / female, n)	85/92	92/80	15/17	132/158	27/15	63/46
	Gestational age (weeks, X ± s)	38.95 ± 2.06	38.40 ± 2.64^*^	36.99 ± 3.48*	39.17 ± 1.75	36.13 ± 3.96^*^	39.04 ± 1.70
	Birth weight (kg, X ± s)	3.20 ± 0.54	3.11 ± 0.64	2.77 ± 0.80^*^	3.23 ± 0.55	2.64 ± 0.94^*^	3.24 ± 0.58
	Birth mode (natural birth / cesarean section, n)	96/81	103/69	15/17	158/132	25/17	50/59
2	Gender (male / female, n)	65/60	57/44	22/23	53/56	34/28	29/21
	Gestational age (weeks, X ± s)	38.44 ± 2.57	38.43 ± 2.54	37.10 ± 3.00*	39.01 ± 1.88	36.21 ± 3.97^*^	38.38 ± 2.22
	Birth weight (kg, X ± s)	3.12 ± 0.65	3.08 ± 0.67	2.56 ± 0.73^*^	3.20 ± 0.54	2.61 ± 0.85^*^	3.14 ± 0.60
	Birth mode (natural birth / cesarean section, n)	64/61	43/58	21/24	47/62	30/32	28/22
3	Gender (male / female, n)	42/27	28/20	26/23	26/24	31/23	12/9
	Gestational age (weeks, X ± s)	37.72 ± 2.81	37.09 ± 3.69	36.68 ± 3.26	37.92 ± 2.66	35.47 ± 4.08^*^	37.59 ± 2.94
	Birth weight (kg, X ± s)	2.93 ± 0.69	2.79 ± 0.89	2.72 ± 0.79	3.04 ± 0.73	2.40 ± 0.91^*^	2.39 ± 1.18
	Birth mode (natural birth / cesarean section, n)	30/39	25/23	22/27	26/24	21/33	9/12
4	Gender (male / female, n)	19/20	16/8	10/12	4/8	14/9	2/2
	Gestational age (weeks, X ± s)	35.20 ± 3.56	34.80 ± 2.97	34.69 ± 3.43	36.88 ± 3.09	34.45 ± 3.48	37.68 ± 3.76
	Birth weight (kg, X ± s)	2.42 ± 0.85	2.21 ± 0.72	2.31 ± 0.77	2.63 ± 0.78	2.17 ± 0.96	2.42 ± 0.28
	Birth mode (natural birth / cesarean section, n)	16/23	11/13	9/13	4/8	10/13	4/0

^*^ compare with normal group *P *< 0.05

### Comparison of TIMP scores of infants with different type of PD at different ages of month

According to the current diagnostic criteria in the infants aged 0 to 6 months, in this research dolichocephaly was the dominant cranial type in the infants aged 0 ~ 1 months. With the increase of month age, the proportion of dolichocephaly and dolicho-plagiocephaly gradually decreased, while the proportion of normal head, brachycephaly and brachy-plagiocephy gradually increased, but the proportion of plagiocephy had no obvious trend of change.

In different groups of PD type, the TIMP scores of 0 and 1 month old dolicho-plagiocephaly infants were significantly lower than those of the normal group, but there was no significant difference between the other rest groups and the normal group. There was also no significant difference in TIMP scores among groups at 2 months of age. In the age groups of 3 months, the TIMP scores of dolichocephaly, dolicho-plagiocephaly and brachy-plagiocephy groups were significantly lower than those of the normal group, and the TIMP scores of thedolicho-plagiocephaly group were the lowest. The TIMP scores of 4 months of age PD groups were lower than those of the normal group, and there were significant differences between the dolichocephaly group and the brachy-plagiocephy group, and the dolichocephaly group had the lowest TIMP scores (Table 3).

**Table 3:** Comparison of TIMP scores of infants with different type of PD at different ages of month

Months of age	n	Plagiocephaly		Brachycephaly		Dolichocephaly		Brachy-plagiocephaly		Dolichol-plagiocephaly		Normal	
		n (%)	TIMP scores	n (%)	TIMP scores	n (%)	TIMP scores	n (%)	TIMP scores	n (%)	TIMP scores	n (%)	TIMP scores
0	389	71 (18.25%)	54.65 ± 6.39	13 (3.34%)	54.85 ± 9.34	145 (37.28%)	54.46 ± 7.61	26 (6.69%)	55.04 ± 6.91	50 (12.85%)	52.54 ± 7.12^*^	84 (21.59%)	55.32 ± 7.00
1	822	172 (20.93%)	59.16 ± 7.83	32 (3.89%)	58.00 ± 6.31	290 (35.28%)	59.55 ± 8.56	42 (5.11%)	60.86 ± 8.42	109 (13.26%)	58.40 ± 8.78^*^	177 (21.53%)	60.69 ± 8.61
2	492	101 (20.53%)	68.32 ± 11.31	45 (9.15%)	65.44 ± 9.78	109 (22.15%)	68.32 ± 12.12	62 (12.60%)	66.58 ± 11.54	50 (10.16%)	68.86 ± 13.61	125 (25.41%)	68.00 ± 11.15
3	291	48 (16.49%)	81.33 ± 17.75	49 (16.84%)	80.04 ± 13.32	50 (17.18%)	74.30 ± 12.34^*#^	54 (18.56%)	76.61 ± 13.70^*^	21 (7.22%)	71.43 ± 13.52^*#^	69 (23.71%)	81.99 ± 13.62
4	124	24 (19.35%)	90.46 ± 13.28	22 (17.74%)	93.45 ± 14.50	12 (9.68%)	82.75 ± 13.03^*△^	23 (18.55%)	87.35 ± 18.78^*^	4 (3.23%)	93.00 ± 4.08	39 (31.45%)	96.92 ± 12.82

^*^compare with normal group *P* < 0.05

^#^compare with plagiocephaly group *P* < 0.05

^△^compare with brachycephaly group *P* < 0.05

### Comparison of TIMP scores of infants with different degrees of PD at different ages of month

#### Comparison of TIMP scores of infants with different degrees of plagiocephy

There was no significant difference in TIMP scores between the plagiocephy group with different degrees and the normal group at the age of 0 month and 2 months, and there was no significant difference within the PD degree groups. The TIMP scores of 1 month old severe plagiocephy group were significantly lower than those of the normal group, mild and moderate plagiocephy groups. The TIMP scores of 3 months old moderate and severe plagiocephy groups were lower than those of the normal group and the mild group, but the difference was not significant. TIMP scores of 4-month-old group with moderate and severe plagiocephy head was significantly lower than those of the normal group and the mildplagiocephy group. (Table 4).

**Table 4:** Comparison of TIMP scores of infants with different degrees of plagiocephy

Months of age	Plagiocephaly								
	n	Mild		Moderate		Severe		Normal	
		n (%)	TIMP scores	n (%)	TIMP scores	n (%)	TIMP scores	n	TIMP scores
0	71	42(59.15%)	55.10 ± 6.51	18(25.35%)	54.28 ± 6.87	11(15.50%)	53.55 ± 5.48	84	55.32 ± 7.00
1	172	104(60.47%)	59.41 ± 7.91	50(29.07%)	60.20 ± 7.15	18(10.46%)	54.83 ± 8.13^*#△^	177	60.69 ± 8.61
2	101	55(55.46%)	68.29 ± 10.37	40(39.60%)	67.40 ± 12.88	6(5.94%)	74.67 ± 6.89	125	68.00 ± 11.15
3	48	28(58.33%)	85.75 ± 17.83	15(31.25%)	75.33 ± 17.87	5(10.42%)	74.60 ± 10.48	69	81.99 ± 13.62
4	24	13(54.17%)	98.54 ± 10.99	3(12.50%)	73.00 ± 14.00^*#^	8(33.33%)	83.88 ± 3.83^*#^	39	96.92 ± 12.82

^*^compare with normal group *P* < 0.0

^#^compare with mild group *P* < 0.0

^△^compare with moderate group *P* < 0.05

#### Comparison of TIMP scores of infants with different degrees of brachycephaly

There was no significant difference in TIMP scores between the brachycephaly group and the normal group, and no significant difference within the different PD degree groups (Table 5).

**Table 5:** Comparison of TIMP scores of infants with different degrees of brachycephaly

Months of age	Brachycephaly								
	n	Mild		Moderate		Mild		Normal	
		n (%)	TIMP scores	n (%)	TIMP scores	n (%)	TIMP scores	n	TIMP scores
0	13	10(76.92%)	53.60 ± 10.27	2(15.38%)	59.00 ± 5.66	1(7.70%)	59.00	84	55.32 ± 7.00
1	32	24(75.00%)	58.88 ± 6.13	8(25.00%)	55.38 ± 6.52	0(0.00%)	/	177	60.69 ± 8.61
2	45	21(46.67%)	65.81 ± 9.24	14(31.11%)	64.57 ± 10.53	10(22.22%)	65.90 ± 8.84	125	68.00 ± 11.15
3	49	26(53.06%)	78.46 ± 12.67	15(30.61%)	85.93 ± 11.61	8(16.33%)	74.13 ± 15.80	69	81.99 ± 13.62
4	22	10(45.45%)	99.50 ± 13.55	10(45.45%)	89.00 ± 13.98	2(9.10%)	85.50 ± 17.68	39	96.92 ± 12.826

^*^compare with normal group *P* < 0.05

^#^compare with mild group *P* < 0.05

^△^compare with moderate group *P* < 0.05

#### Comparison of TIMP scores of infants with different degrees of dolichocephaly

The TIMP scores of 0 month old severe dolichocephaly group were significantly lower than those of the normal group, and the scores of moderate and severe dolichocephaly groups were significantly lower than those of the mild dolichocephaly group. There was no significant difference in TIMP scores between different degrees of dolichocephaly and normal groups at 1 and 2 months of age, as well as no significant difference within the different degree groups. The TIMP scores of 3 months old group with severe dolichocephaly were significantly lower than those of the normal group, mild and moderate dolichocephaly groups. The TIMP scores of 4 months old group with moderate and severe dolichocephaly were significantly lower than those of the normal group. (Table 6).

**Table 6:** Comparison of TIMP scores of infants with different degrees of dolichocephaly

Months of age	Dolichocephaly								
	n	Mild		Moderate		Mild		Normal	
		n (%)	TIMP scores	n (%)	TIMP scores	n (%)	TIMP scores	n	TIMP scores
0	145	53(36.56%)	57.17 ± 7.86	46(31.72%)	53.54 ± 6.71^#^	46(31.72%)	52.24 ± 7.36^*#^	84	55.32 ± 7.00
1	290	116(40.00%)	59.26 ± 8.34	105(36.21%)	59.74 ± 8.40	69(23.79%)	59.74 ± 9.24	177	60.69 ± 8.61
2	109	39(35.78%)	66.10 ± 10.67	39(35.78%)	69.49 ± 12.70	31(28.44%)	69.65 ± 13.07	125	68.00 ± 11.15
3	50	18(36.00%)	75.72 ± 10.73	24(48.00%)	75.50 ± 12.86	8(16.00%)	65.50 ± 10.56^*#△^	69	81.99 ± 13.62
4	12	4(33.33%)	88.75 ± 11.62	5(41.67%)	84.20 ± 12.99^*^	3(25.00%)	72.33 ± 12.42^*^	39	96.92 ± 12.826

^*^compare with normal group *P* < 0.05

^#^compare with mild group *P* < 0.05

^△^compare with moderate group *P* < 0.05

### Comparison of PD in infants with different levels of TIMP scores at different ages of month

#### Comparison of CVA and CI values of infants with different TIMP score levels

The CVA values of infants in the extremely low TIMP score level group were higher than those in the medium and high level groups at all months of age, but the difference was not significant at 0–2 months of age. At 3 months of age, the CVA values of the infants in the extremely low-level group were significantly higher than those in the medium and low-level groups. At 4 months of age, the CVA values of the infants in the extremely low-level group and low-level group were significantly higher than those in the medium and high-level groups. There was no significant difference in CI values of different TIMP score levels between groups at 1, 2 and 4 months of age. The CI values of the infants at 0 months of age in the extremely low-level group were the lowest in all groups, which was significantly different from those in the high-level group. The CI values of the infants at 3 months of age in the low-level group were the lowest and significantly lower than those in the medium and high-level groups (Table 7).

**Table 7:** Comparison of CVA and CI values of infants with different TIMP score levels

Months of age	n	TIMP scores											
		Extremely low-level			Low-level			Medium-level			High-level		
		n(%)	CVA	CI	n(%)	CVA	CI	n(%)	CVA	CI	n(%)	CVA	CI
0	389	7 (1.80%)	3.86 ± 3.58	78.80 ± 7.08^#^	24 (6.17%)	3.88 ± 4.24	82.45 ± 7.02	254 (65.30%)	3.60 ± 2.90	82.34 ± 5.77	104 (26.73%)	3.22 ± 2.68	83.48 ± 6.06
1	822	27 (3.28%)	4.25 ± 3.54	80.55 ± 5.47	112 (13.63%)	3.83 ± 3.15	82.31 ± 5.87	470 (57.18%)	3.47 ± 2.65	82.93 ± 5.76	213 (25.91%)	3.39 ± 2.54	82.73 ± 5.63
2	492	43 (8.74%)	4.23 ± 3.18	85.92 ± 6.78	132 (26.83%)	3.90 ± 2.97	85.60 ± 6.91	239 (48.58%)	3.71 ± 2.78	85.68 ± 6.98	78 (15.75%)	3.95 ± 2.63	84.49 ± 5.95
3	291	48 (16.49%)	5.04 ± 3.81^*△^	87.88 ± 8.62	75 (25.77%)	3.36 ± 2.44	85.10 ± 7.21^*#^	137 (47.08%)	3.85 ± 3.20	88.28 ± 6.61	31 (10.66%)	3.94 ± 2.14	88.82 ± 5.41
4	124	36 (29.03%)	4.86 ± 4.43^*#^	89.59 ± 8.65	23 (18.55%)	6.30 ± 4.90^*#^	88.50 ± 6.41	51 (41.13%)	3.24 ± 2.29	88.52 ± 5.63	14 (11.29%)	2.64 ± 1.73	90.61 ± 5.33

^*^compare with medium group *P* < 0.05

^#^compare with high group *P* < 0.05

^△^compare with low group *P* < 0.05

#### Comparison of PD types of infants with different TIMP score levels

At 0–1 month of age, all groups of TIMP score level of infants were dolichocephaly. At 2 months of age, the extremely low-level group had the highest proportion of plagiocephy, and also had the highest proportion of brachy-plagiocephy at 3 and 4 months of age, while the medium-level and high-level groups had the highest proportion of normal head type (Table 8).

**Table 8:**
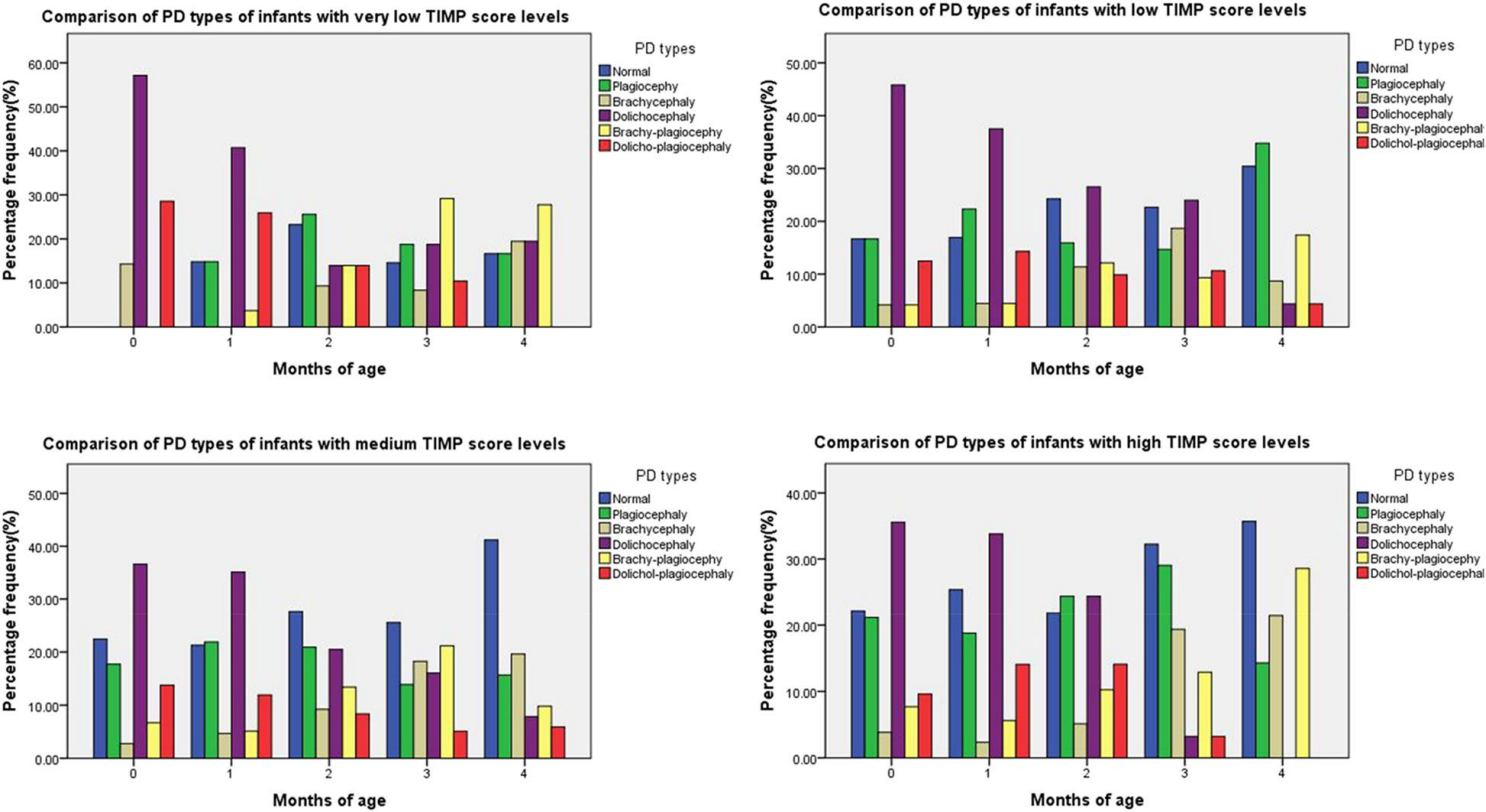
Comparison of PD types of infants with different TIMP score levels

## Discussions

PD is one of the common situations in early infancy. In the months after the birth of infants, PD often occurs due to the fast growth of the brain, low hardness of skull, existence of fontanel and cranial sutures, and poor head control resulting in fixed head posture [19, 20]. In addition to fixed postnatal sleeping position, craniotypic abnormalities in infants were also related to premature birth, birth weight, male and other factors [1]. Analysis of general data in this research showed differences in gestational age and birth weight between Infants with PD and normal infants,but there seems to be no significant difference in gender and mode of delivery, which is different from the results of LinzC et al.It should be further explored in future research.

PD has not been paid enough attention over a long period of time in China, mainly because most people consider that PD are only a small regret that merely affects children's craniotypic appearance and will not affect children's motor development. Actually, this issue has been discussed by some scholars in the world. Hussein MA et al. used The Bayley Scales of Infant and Toddler Development- ii (BSID- ii) to evaluate 155 PD children aged 4 to 36 months. It was found that the mean value of psychomotor development index (PDI) of them was lower than that of normal children (92.28 ± 17.6 vs 100 ± 16) [21]. Fontana SC et al., used The Bayley Scales of Infant and Toddler Development- iii (BSID- iii) to evaluate 27 infants with PD aged from 4 to 11 months, found that infants with PD,22% had retardation of motor development [22]. Moreover, Zhao Xue-Qing et alalso evaluated 393 children with postural plagiocephy from 0 to 18 months with Infant Neurological International Battery (INFANIB), and found that the INFANIB scores of children with postural plagiocephy were significantly lower than those of normal children. It was considered that the reasons for the differences might be related to more postural abnormalities and asymmetries in children with plagiocephy [23]. All these studies suggested that PD was associated with motor development in children, however, most of these studies foucused on the children with cerebral palsy and other neurodevelopmental abnormalities. Also, the subjects studied in these researches were mostly older than 4 months of age, an age when infants are less likely to have PD than in previous months due to deceleration of brain growth, increased skull stiffness, and improved ability of head control. Therefore, the ideal time to explore the interaction between PD and the delay of the motor development should be considered before 4 months of age [12, 24].

Nonetheless, it is difficult to assess the motor development of infants less than 4 months of age. The age span of most of the global developmental assessment tools used at present is wide, usually between 0-5 years or more, and there are few assessment items in these tools for infants younger than 4 months old, which affects the ability of these tools to detect the exercise level of infants at this age [25, 26]. TIMP is currently the most widely used tool for early motor performance assessment of infants before 4 months of age in the world, and the Chinese version of the norm was revised after it was officially introduced into China in 2017 [27]. At present, there are many international reports on the identification of diseases through TIMP that can lead to motor development retardation in infants, including cerebral palsy, Spinal Muscular Atrophy (SMA), Down syndrome and so on [28–30]. From the perspective of the causes of PD, PD is more related to reduction of movement and fixation of posture in infants. Therefore, it is a reasonable choice that the Chinese version of TIMP norm was used as an indicator to evaluate and group the motor performance level of infants under the age of 4 months.

In this study, it was found that PD were indeed correlated with infants' early functional motor development.Especially in the 3–4 month age group, dolichocephaly, plagiocephaly, dolicho-plagiocephaly and brachy-plagiocephy had significant effects on infant motor development, while brachycephaly had no significant effects..We presume that the poor movement of infants with dolichocephaly might be related to the excessive sleeping posture in the lateral position. The lateral position has certain restrictions on the symmetry of the midline position, supine position maintenance and limb movement ability of infants, resulting in the low TIMP scores. Also, the ability of maintaining symmetry in the midline position of infants with plagiocephaly was limited due to long periods of asymmetric sleeping position which resulted in the decline of TIMP scores. Furthermore, we found that the more serious the severity of dolichocephaly and plagiocephaly, the more significant influence on motor development of infants. Meanwhile, infants with dolicho-plagiocephaly and infants with brachy-plagiocephy, both of which suffered from a combination of two types of skull deformation (having both dolichocephaly and plagiocephy, or both brachycephaly and plagiocephy), had more limited gross motor function and lower TIMP score. However Infants with brachycephaly were more likely to maintain the head control in the midline position because they had long periods of midline supine position in sleeping and thus scored easily in related TIMP items.

On the other hand,the infants in this study were divided into different level groups based on the percentile grade of TIMP scores, and we focused on cranial type changes in infants in the extremely low-level group below the 10th percentile. The results showed that the CVA values of infants in the extremely low-level group (< P10) were larger than those in the medium-level and high-level groups at the whole sequence of the month age, while the CI values in different level groups had no significant difference. Therefore, it can be concluded that poor motor performance was primarily associated with an increased risk of plagiocephy in infants. Furthermore, the proportion of infants with brachy-plagiocephy in the very low level group of TIMP at 3–4 months old was significantly higher, while the proportion of infants with normal cranial type in the middle and high level group of TIMP was the higher. This suggested that infants with normal motor development were more likely to have normal cranial type, while in infants with low-level of motor performance, the most probable PD type was brachy-plagiocephy. The reason may be that infants with poor motor performance had limited ability of anti-gravity movement in head control and position switching, so they were more likely to maintain a certain fixed sleeping position, resulting in PD. On the contrary, the infants with better motor performance were more likely to have better head control and autonomous head rotation in younger age, which could avoid the continuous compression of partial area of skull in the lying position, so the normal cranial type was in the majority of these infants.

## Conclusions

According to the statistical analysis and results,PD and motor performance of infants seem to interact and influence each other. Therefore,PD should not be treated as a purely cosmetic problem, but rather a concern about the motor development in children with PD. It should be noted, however, that our findings only suggest a correlation between PD and TIMP score before 4 months of age, and may not indicate that infants with PD will also have lower motor performance later in older age through the neurodevelopment stage. The long-term motor performance outcomes of infants with PD needs to be followed up in longitudinal studies with considering of other motor-development-related influencing factors. Still, the results of this study suggest that in early infancy severe plagiocephy or brachy-plagiocephy can be an early warning signal of poor motor development in infants and need to be paid attention.

## Limitation and future directions

The results of this study should be considered in light of the following four limitations. Firstly, the longitudinal follow-up study of infant neurodevelopmental status has not been completed yet, and at present PD can only be identified as a risk factor for motor development delay and rather a prediction in the long term outcome. Secondly, the information on socio-economic status (SES) of the infant’s family was not provided, future studies could embrace the variables in the following-up investigation in order to verify confounding factors that are unmeasured. Thirdly, as the frequency of returning to the hospital gragually decreased with the increase of infant age, the drop out had also increased, resulting in the small number of cases in some groups, which may lead to bias of the results. Furthermore, since there is no unified diagnostic standard for PD in China, we adopted our own early diagnostic suggestions for diagnosis and grading. It shoud be noted that the cranial shape of infants is generally ‘long-head’ soon after the birth, and with the increase of supine position time, there is a overall trend showing that cranial shape changes from long to flat. This suggests that the recommended criteria for PD should be more precise in terms of monthly age, or even weekly age, otherwise it may lead to over diagnosis of dolichocephaly in the first 2 months of infancy. We will continue to work with several domestic medical institutions to collect data on the head shape of infants in different regions of China, and hope to have a more optimized reference standard in the near future.

## Data Availability

Authors can confirm that all relevant data are included in the article and,The authors declare that [the/all other] data supporting the findings of this study are available within the article.

## Abbreviations

PD: Positional skull deformation
DP: Deformational plagiocephaly
DB: Deformational brachycephaly
PP: Positional plagiocephaly
CVA: Cranial vault asymmetry
CI: Cephalic index
TIMP: The infant motor performance test
DA: The long diameter is diagonal A
DB: The short diameter is diagonal B
BSID- ii: The Bayley Scales of Infant and Toddler Development- ii
MDI: The mean mental development index
PDI: The mean value of psychomotor development index
BSID- iii: The Bayley Scales of Infant and Toddler Development- iii
INFANIB: Infant Neurological International Battery
SMA: Spinal Muscular Atrophy
SES: Socio-economic status
